# Bibliometric analyses of factors influencing color preferences in urban environmental spaces

**DOI:** 10.3389/fpsyg.2025.1588644

**Published:** 2025-05-09

**Authors:** Chunshuo Qu, Jianfei Dong, Weijia Wang

**Affiliations:** ^1^School of Architecture and Design, Harbin Institute of Technology, Harbin, China; ^2^Key Laboratory of Cold Regions Urban Rural Human Settlement Environment Science and Technology Ministry of Industry and Information Technology, Harbin, China

**Keywords:** urban space environment, color preference, environmental psychology, color psychology, bibliometric analyses

## Abstract

Urban color is a crucial visual component of urban aesthetics, directly reflecting national traditional aesthetic consciousness and historical culture, and holding significant aesthetic value and research relevance. As a key factor in urban color studies, color preference significantly impacts human behavior and plays a vital role in urban planning and development. However, a comprehensive and systematic understanding of urban color preferences remains underexplored. This study examines 379 relevant studies using bibliometric review methods and literature visualization tools, classifying, and synthesizing them according to research hotspots and fields. Four primary categories influencing urban color preference were identified: personal attributes, socio-cultural influences, environmental space, and dynamic factors. The analysis also highlights the psychological mechanisms underlying color preference, including its effects on emotional regulation, cognitive processing, and social identity. The findings contribute to the field by offering a data-driven framework for understanding urban color preferences and proposing practical applications for urban planning. The study emphasizes the importance of color preference in promoting human well-being and sustainable urban development, with implications for future interdisciplinary research.

## 1 Introduction

In the early 21st century, the gradual development of countries reached a zenith in urban construction, accompanied by a growing interest in the use of color in cities. Color is one of the primary visual dimensions of an environment (Kotler, 1974) and an important factor affecting people’s overall evaluation of a city. When individuals are presented with information, color plays a significant role in their perceptions during the initial 20 s, comprising 80% of the total (Livingstone, 2008; Yao et al., 2022). Therefore, color is the primary element that affects the visual quality of a city, strongly stimulating people’s visual perception and shaping their impressions of the urban environment.

Different cities have developed their own distinctive color systems shaped by their unique natural environments, climatic conditions, and cultural backgrounds. These color systems not only reflect regional cultural characteristics but also contribute to the visual identity of urban landscapes (Xing and Guo, 2024). As urban color research continues to deepen, scholars increasingly recognize that color is not only an important part of urban aesthetics, but also plays a significant role in shaping spatial perception (Wan et al., 2020), enhancing emotional experience (Wang et al., 2022), fostering social interactions (Buechner et al., 2015), and influencing behavioral responses (Ren and Chen, 2018). Therefore, the creation of color schemes in urban environments that positively influence residents’ emotional and behavioral responses, along with the exploration of how urban color systems are formed and how they affect human behavior, has become a significant research direction.

When studying the factors influencing urban color preferences, it is essential to analyze not only macro-level aspects such as urban composition and land-use planning but also to explore the emotional responses and psychological needs of residents in their daily lives and work. By optimizing color design, it is possible to enhance the visual appeal of urban environments and improve residents’ sense of well-being and life satisfaction, thereby promoting the development of more livable and harmonious cities.

In recent years, the number of studies on urban color preferences has gradually increased, encompassing a range of dimensions including the natural environment, cultural traditions, social psychology, and policy frameworks. For instance, considerable attention has been devoted to the impact of climatic conditions on color selection—with colder regions generally favoring warm tones (Azer, 2020), while tropical regions prefer cooler shades to mitigate the sensation of heat. Additionally, socio-cultural factors such as religious beliefs, historical traditions, and national customs significantly influence urban color preferences (Lenclos and Lenclos, 2004). However, most existing studies focus on a single factor or specific region, lacking a comprehensive exploration of the interactions between multiple dimensions. As a result, the mechanisms behind color preferences in complex urban spatial contexts remain insufficiently understood.

Building upon this background, the present study aims to systematically identify the key factors influencing urban color preferences through an extensive and critical review of the existing literature. It further investigates the complex interplay among natural, cultural, psychological, and socio-environmental dimensions that shape color perception within urban spatial contexts. In addition, the study evaluates the methodological frameworks and thematic focuses of current research to uncover commonly observed limitations and knowledge gaps. By establishing a conceptual framework for understanding the mechanisms of urban color preferences, this research ultimately seeks to provide evidence-based insights and practical guidance for urban planners, designers, and policymakers in developing emotionally responsive and context-sensitive urban color systems.

## 2 Methods

### 2.1 Headings manuscript

**Figure 1:**
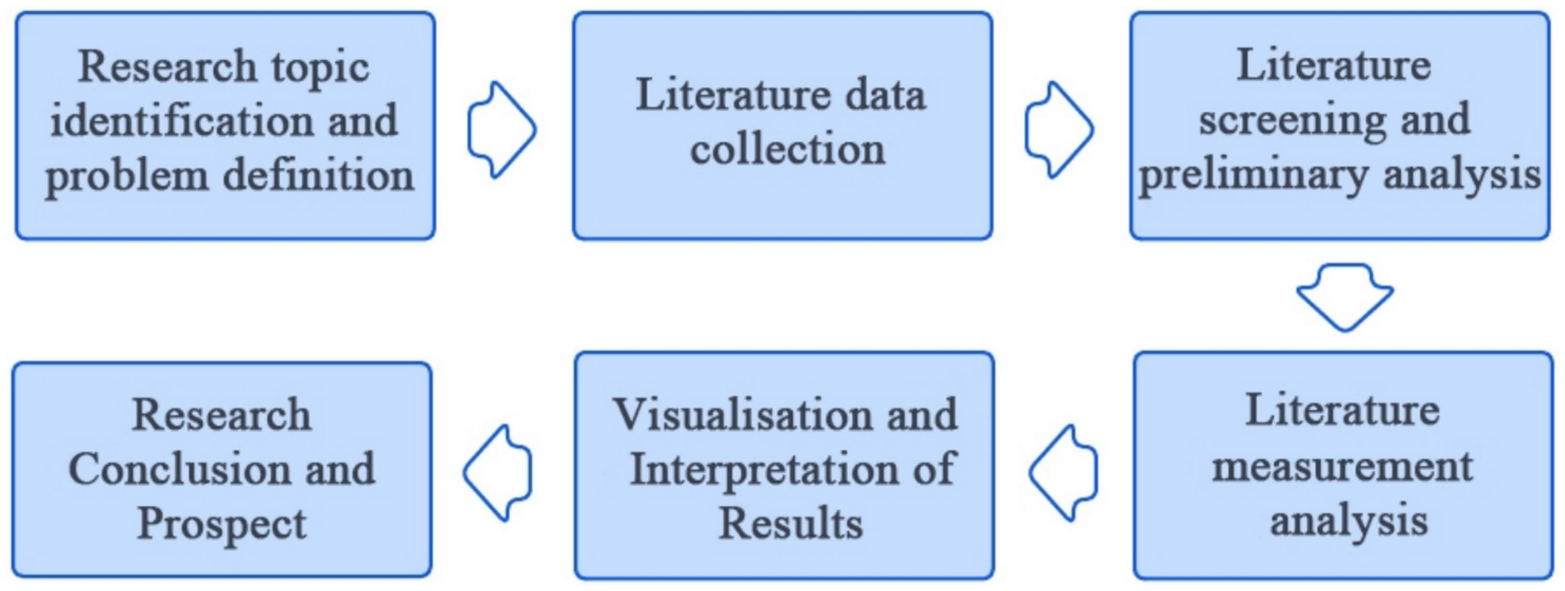
Steps of the bibliometric study.

This study employs the bibliometric method to systematically review and analyze the current research status and development trend in the field of factors influencing spatial color preferences in urban environments. Bibliometrics can objectively reveals the knowledge structure, research hotspots, and evolution of the field through quantitative analysis and visual representation of elements such as publication volume, sources, institutions, authors, keywords, and citation networks, etc., so as to provide scientific basis and theoretical support for the subsequent research (Figure 1).

To ensure that the literature data were comprehensive, authoritative, and of high impact, this study identified the primary data sources. The search strategy incorporated a combination of subject terms and keywords with Boolean logic operators to enhance both accuracy and comprehensiveness (Aria and Cuccurullo, 2017). Additionally, the search timeframe was selected to capture the evolution of the field and highlight its latest advancements.

In order to reveal the knowledge and evolution path of the research field in a more systematic and in-depth way, this study employs CiteSpace for bibliometric visualization analysis. The core themes and research hotspots were identified through co-occurrence analysis, while co-citation analysis was used to trace the origins of knowledge and assess academic influence. Additionally, collaborative network analysis was conducted to map research teams and their cooperative relationships. Furthermore, cluster analysis was applied to summarize key factors influencing color preferences in urban environments and to explore their underlying associations and mechanisms. Finally, based on the chronological analysis of research hotspots, the development stages, key issues and future trends of the field are summarized to provide systematic support for subsequent theoretical research and empirical analysis.

### 2.2 Data sources

The literature data of this study was obtained from the Web of Science (WoS) Core Collection, which encompasses high-impact, rigorously peer-reviewed academic journals on a global scale with high authority and broad subject coverage. It also provides a substantial number of high-quality, widely cited journal articles, conference papers, books, and other resources, which can provide a reliable literature base for the present study. It also provides a large number of high-quality and widely cited journal articles, conference papers, books and other resources, which can provide a robust and reliable foundation for this research (Li et al., 2018; Tian and Liu, 2024).

In order to ensure the standardization of literature screening and management, this study used EndNote Literature Management Software to systematically organize all the included literature, including literature import and de-weighting; classification and labelling of literature information; and standardization of citation format. By implementing a rigorous data sourcing and screening process, this study ensures the comprehensiveness, scientific rigor, and high quality of the literature review, providing a solid literature base for subsequent analyses and discussions.

### 2.3 Data filtering

This review followed the three-stage review methodology of identifying (Petticrew and Roberts, 2005), analysing, and interpreting and followed the literature review guidelines where applicable (Moher et al., 2009). A comprehensive search was conducted using the Web of Science Core Collection database. The search formula was as follows: TS = (“urban color” OR “urban color” OR “city color” OR “architectural color” OR “urban space color”) AND (“preference” OR “perception” OR “emotion” OR “identity” OR “culture”).

The search was limited to publications between 1985 and May 2024, and only articles and review papers written in English were included. A keyword search for urban/city color /color preference/urban environment color preference revealed a total of 13,037 studies from 1985 until May 2024.

After removing duplicates, non-English literature, and non-“research articles,” as well as screening based on title, keywords, and abstracts, a total of 848 studies were excluded. During this screening process, non-core journal papers and papers unrelated to the review question were excluded.

**Figure 2:**
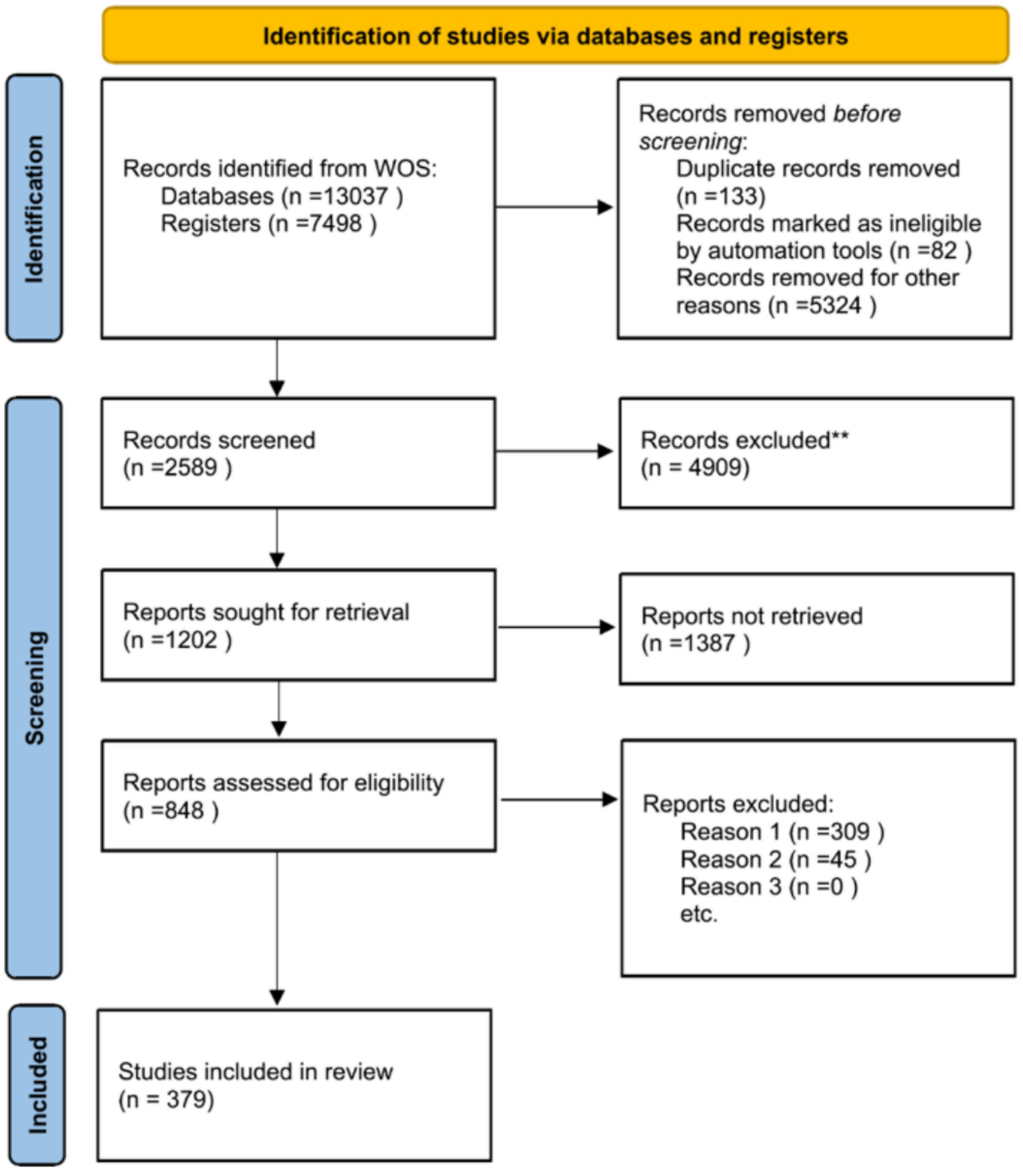
Flowchart on literature search, screening, and selection.

Eligible papers were reviewed in detail, and data related to the above questions were extracted. In addition, selected bibliographies of the included papers were reviewed to supplement the review database. After reviewing the collected studies, 379 relevant publications were deemed eligible for inclusion in this study (Figure 2). However, given the space limitations of journal articles, only a selected number of the most relevant studies were cited. Due to resource constraints, this paper does not cover all relevant publications. The findings of the reviewed studies are categorized based on their subject matter to identify research gaps and areas that have received limited attention.

### 2.4 Keywords clustering and co-occurrence

To identify the core themes and research trends in the study of color preferences in urban environments, this paper first analyses the publication patterns of the reviewed studies, including the number of publications, geographical distribution, affiliated institutions, and research teams, using CiteSpace 6.2.2 software (Li and Chen, 2006).

The study covers the period from 1985 to 2024, and the data exported in .txt format, include titles, abstracts, keywords, and references. In CiteSpace, the time slicing parameter is set to 5 years, the TopN threshold is set to 50, and all other threshold values remain unchanged. To validate the mapping results, module values (*Q*-values) and average silhouette values (*S*-values) are generated in CiteSpace to assess their frequency of use and distribution in the literature.

The *Q*-value ranges from [0, 1), where a *Q*-value greater than 0.3 indicates a more significant cluster structure. The *S*-value ranges from [−1, 1], and when the *S*-value exceeds 0.5, the clusters are considered well-defined; a higher *S*-value indicates greater homogeneity within the clusters. Meanwhile the time axis changes from far to near, and the color changes from bright to bland. The core of the research field is assessed using mediated centrality.

## 3 Results

### 3.1 Publication and distribution

**Figure 3:**
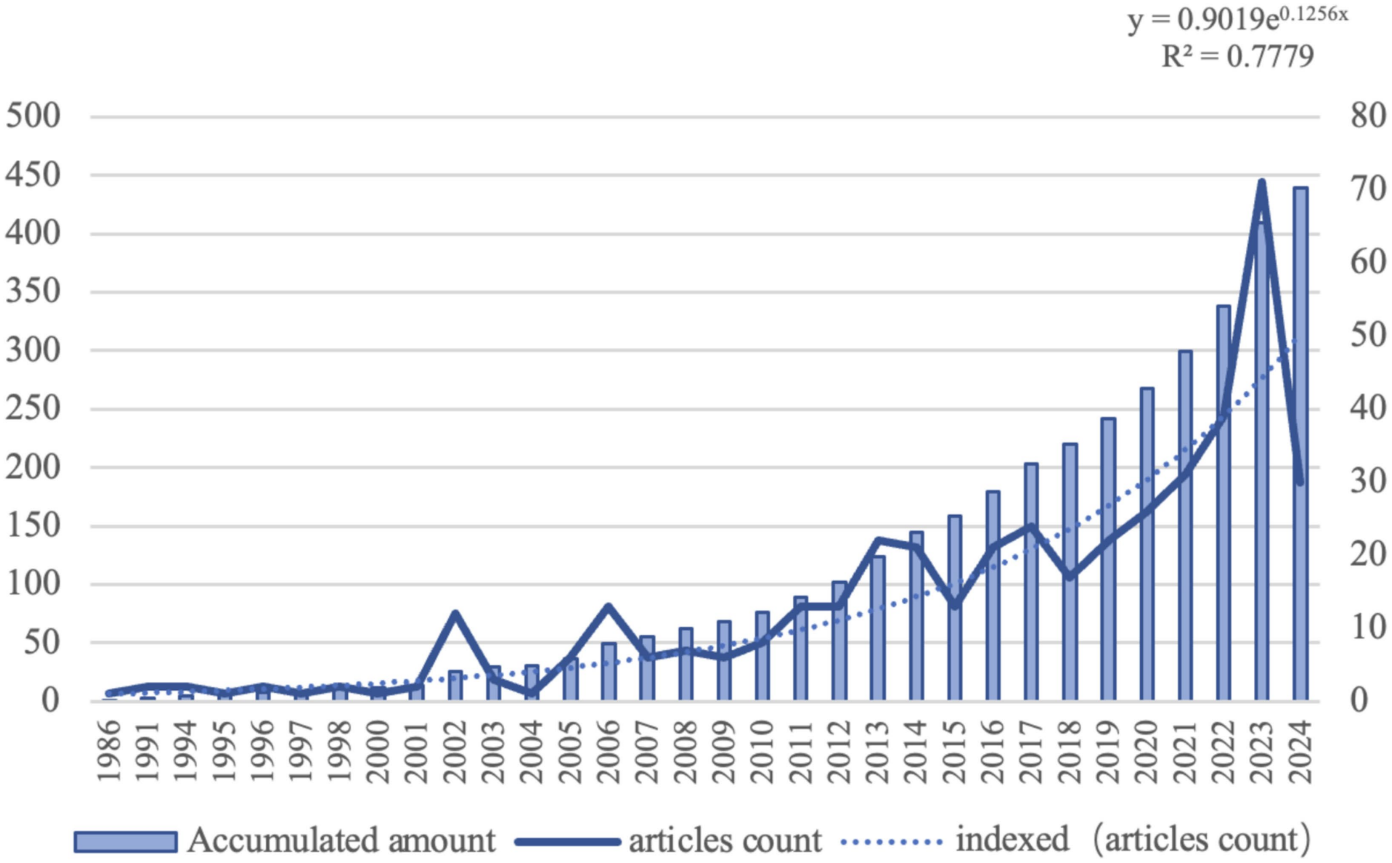
Number of urban color articles published by year (1985–2024).

The number of published articles serves is to some extent, as an indicator of the current state of research and emerging trends in this field. Specifically, this trend reflects a growing concern for the quality of the visual environment and an increased demand for color. Figure 3 illustrates that the overall trajectory of research on urban color preferences has been on an upward trend, especially peaking in 2023.

**Figure 4:**
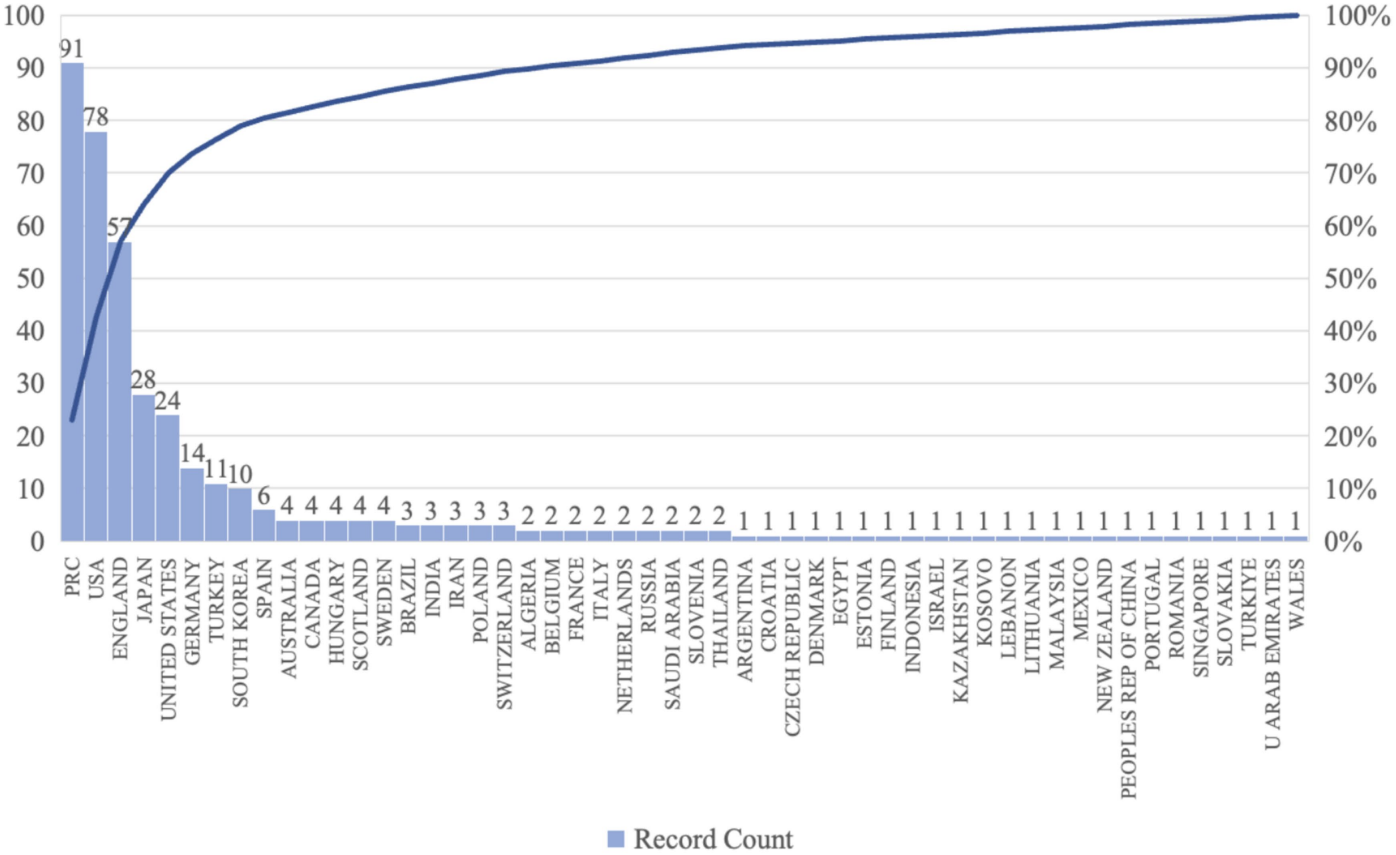
National and regional research subdivisions.

The spatial distribution of urban color preference research in different countries is shown in Figure 4, the research has been carried out in 52 countries and regions in all continents except Antarctica, of which 18 cities are in Asia, 23 are located in Europe, and there are fewer participating countries in North America and Africa. Among them, China is at the top of the list with a total of 91 articles since 2001, and the United States of America is at the second place in terms of the number of articles published, and the first research on the preference of urban color was proposed in 1985, with a total of 78 articles published since then.

### 3.2 Analysis of visual network

**Figure 5:**
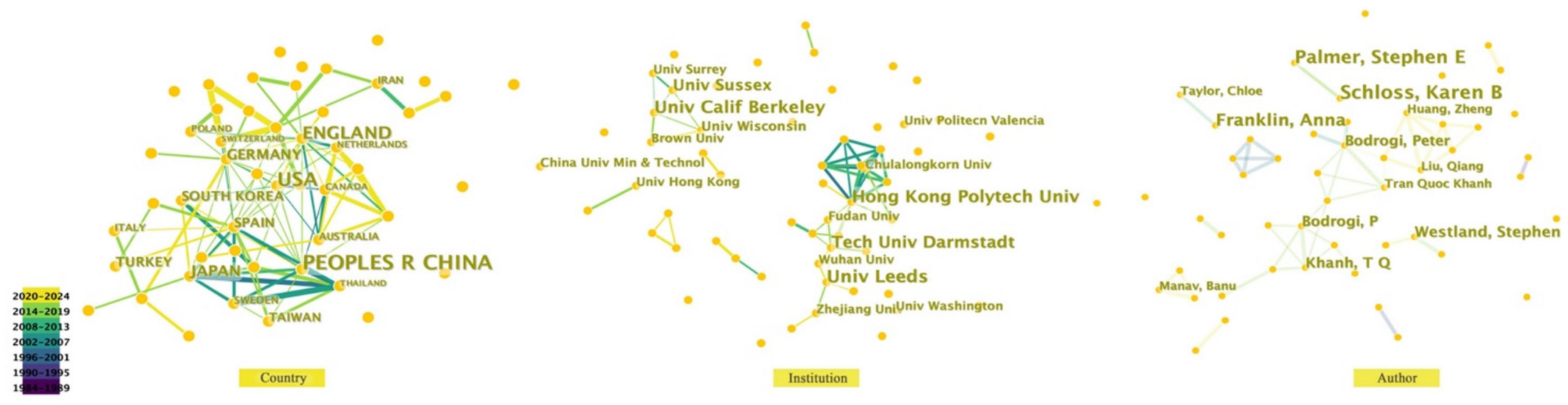
Distribution of countries, regions, institutions, and authors.

Regarding research institutions and teams (Figure 5), the University of Leeds, the University of California, Berkeley, and Darmstadt University of Technology are leading the way in the study of urban color preferences. The University of Leeds has published the most papers between 2020 and 2024, with 13, and its research team focused on the impact of color combination preferences. The primary study period for the University of California at Berkeley was 2016–2024, and they published a total of 12 papers. The re-search team proposed that color preference is based on people’s average emotional responses to color-related objects. The Darmstadt University of Technology was active from 2016 to 2024, publishing 11 articles. The team focused on the influence of urban light sources on color preferences.

**Figure 6:**
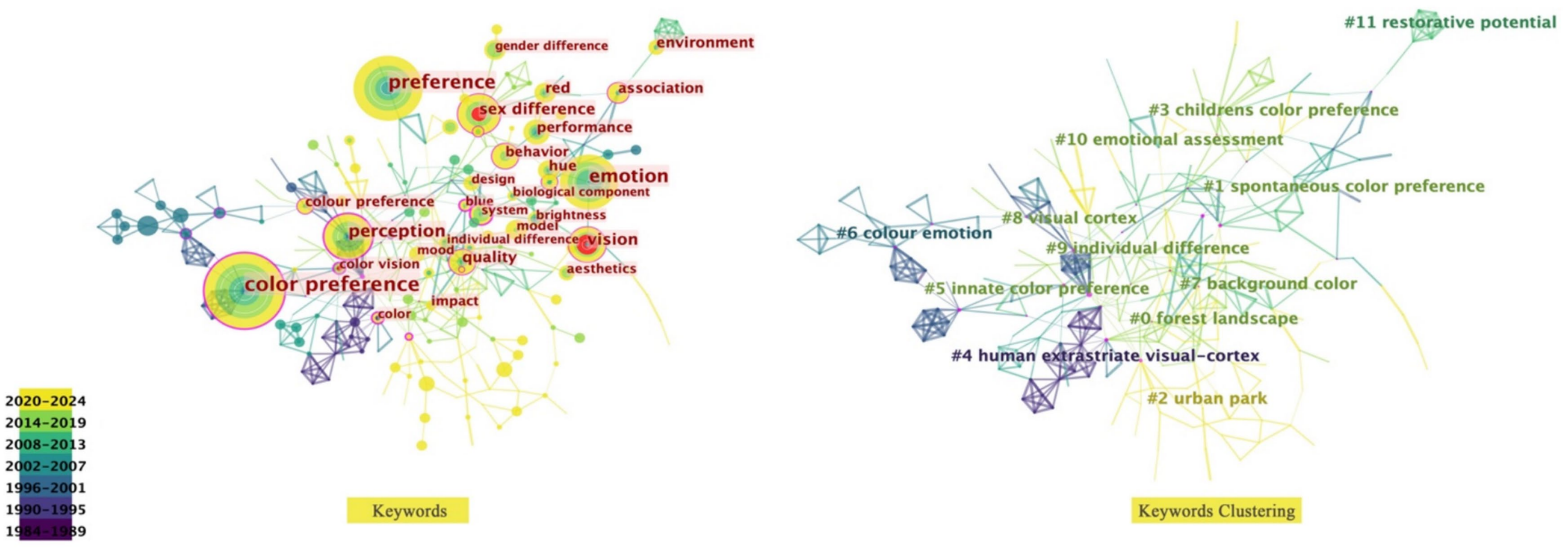
Keyword co-occurrence mapping and cluster analysis.

Subsequently, the analysis is based on the topic of the researched paper. According to the co-occurrence analysis of keywords, the research development hotspots, current status and future research trends in urban color preference research can be drawn. The most frequently researched areas are color preference, preference, and emotion; visual aesthetics has become the focus of urban color preference research, and visual research on vision increased dramatically in 2001. Since 2013, research on the difference in color preference between men and women has increased dramatically, and the topic of sex difference has become a keyword in that year (Figure 6).

A total of 11 clusters can be obtained through keyword clustering, which include forest landscape, spontaneous color preference, urban park, children’s color preference, human *in vitro* visual cortex, innate color preference, color emotion, environmental color, visual cortex, individual differences, external color, and recovery potential. These clusters represent the main research fields of urban color preference.

**Figure 7:**
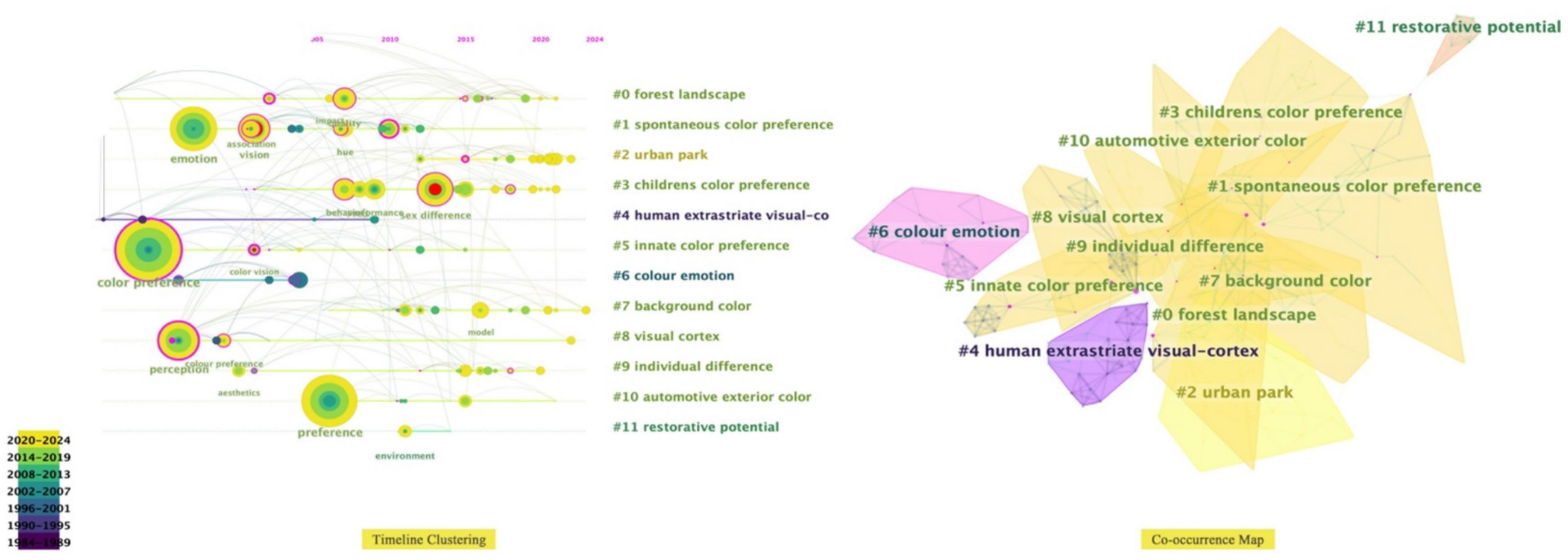
Keyword clustering timeline.

A temporal analysis of keyword usage in published articles (Figure 7) reveals that research on urban color preference is concentrated in the period 2015–2023. This represents a significant focus of recent urban research. Over the past 9 years, there has been a growing recognition of the challenges posed by human activity. The number of topics related to humans reached its peak in 2016, including research on the human visual system. Other related topics that have been mentioned in the articles under study have attracted less attention.

Each of these 11 clustered themes has been extensively studied over the past 38 years. Among these, themes associated with environmental sciences have generated the most academic discussion, primarily focusing on visual quality (Arriaza et al., 2004; Du et al., 2016; Zhao et al., 2017), environmental features (Pazhouhanfar and Shariff, 2014; Li et al., 2019), and natural ecology (Lee et al., 2014). The second cluster pertains to behavioral sciences, which examine individual variables in relation to urban color preference behaviors (Fernandez-Cañero et al., 2013). The third cluster is the psychological sphere, emphasizing the factors underlying individuals’ color preference choices (Todorova et al., 2004; Kardan et al., 2015). Additionally, the effect of place change has emerged as a prominent research topic in recent years, despite a relatively little number of publications. The analysis of the most frequently indexed articles aids in identifying research hotspots (see Table 1).

**Table 1:** Presents the top 10 most frequently cited articles in urban color preferences.

No.	Title	Author	Year	Journal	Citation
1	An ecological valence theory of human color preference (Palmer and Schloss, 2010)	Stephen E. Palmer, Karen B. Schloss	2011	Proceedings of the National Academy of Sciences	487
2	Visual aesthetics and human preference (Palmer et al., 2013)	Stephen E. Palmer, Karen B. Schloss, Jonathan Sammartino	2013	Annual Review of Psychology	441
3	Managing images in different cultures: a cross-national study of color meanings and preferences (Madden et al., 2000)	Thomas J. Madden, Kelly Hewett, Martin S. Roth	2000	Journal of International Marketing	352
4	A study of color emotion and color preference. Part I: color emotions for single colors (Ou et al., 2004a)	Li-Chen Ou, M. Ronnier Luo, Andrée Woodcock, Angela Wright	2002	Color Research & Application	387
5	An ecological valence theory of human color preference (Palmer and Schloss, 2010)	Stephen E. Palmer, Karen B. Schloss	2017	Vision Research	362
6	Biological components of sex differences in color preference (Hurlbert and Ling, 2007)	Anya C. Hurlbert, Yazhu Ling	2007	Current Biology	266
7	Innate color preferences of flower visitors (Lunau and Maier, 1995)	Klaus Lunau, E. J. Maier	1995	Journal of Comparative Physiology A	225
8	Children’s emotional associations with colors (Boyatzis and Varghese, 1994)	Chris J. Boyatzis, Reenu Varghese	1994	The Journal of Genetic Psychology	212
9	A study of color emotion and color preference. Part III: color preference modeling (Ou et al., 2004c)	Li-Chen Ou, M. Ronnier Luo, Andrée Woodcock, Angela Wright	2004	Color Research & Application	176
10	A system of color-preferences (Guilford and Smith, 1959)	J. P. Guilford, Patricia C. Smith	1959	The American Journal of Psychology	173

Highly-frequency cited literature indicates that color preferences are influenced by multiple factors, including ecological potency, emotional responses, cultural differences, gender and age differences, and evolutionary instincts. Individuals tend to prefer colors associated with positive experiences and show distinct preference patterns depending on cultural background and gender and age differences. Furthermore, color preferences can be systematically expressed through models and associated with variables such as affective responses, thereby providing a theoretical for quantitative analysis in color preference research.

## 4 Discussion

It is found that among the research on urban color preference, there are more studies on the scope of human psychology, of which the top three cited papers are visual quality research, emotion and color preference, and the theory of human color preference. Color preference was the earliest concepts proposed in studies on color (Hurlbert and Ling, 2007; Birren, 1961). Color environments exert a profound effect on individuals’ behavioral and emotional preferences. In this paper, the factors of color preference are classified into individual factors (Ou et al., 2004a, 2004b), socio-cultural factors (Jameson and Webster, 2019), environmental factors (Li and Cao, 2022), and dynamic factors (Schloss et al., 2011) to describe the mechanisms and methods by which urban color preferences are shaped.

### 4.1 Individual attributes and psychological cognition of color

Individual factors significantly influence urban color preferences, particularly in terms of emotional, psychological, and personal experiences (Wright and Rainwater, 1962; Elliot et al., 2016).

Human color preferences are influenced by both age and gender to varying degrees. Two-month-old infants develop some behavioral response to color stimuli infants prefer wavelengths of the spectrum (Teller et al., 1978), with red and blue being perceived highly, but blue is generally preferred (Bornstein, 1975; Zemach et al., 2007). By the age of 3 months, infants begin to exhibit spontaneous color preferences (Zemach and Teller, 2007), and these preferences tend to continues into adulthood (Hartzler et al., 2010). Infants exhibit a preference for wavelengths of the spectrum in which red and blue are perceived highly (Bornstein, 1975; Zemach et al., 2007). This also contributes to an innate predisposition for color preference. At this stage, the color characteristics of the visual environment play an important role in shaping an infant’s color preferences (Franklin et al., 2010). Consequently, the quality of color design in an individual’s early-life environment influences both color choices and psycho-cognitive development.

**Figure 8:**
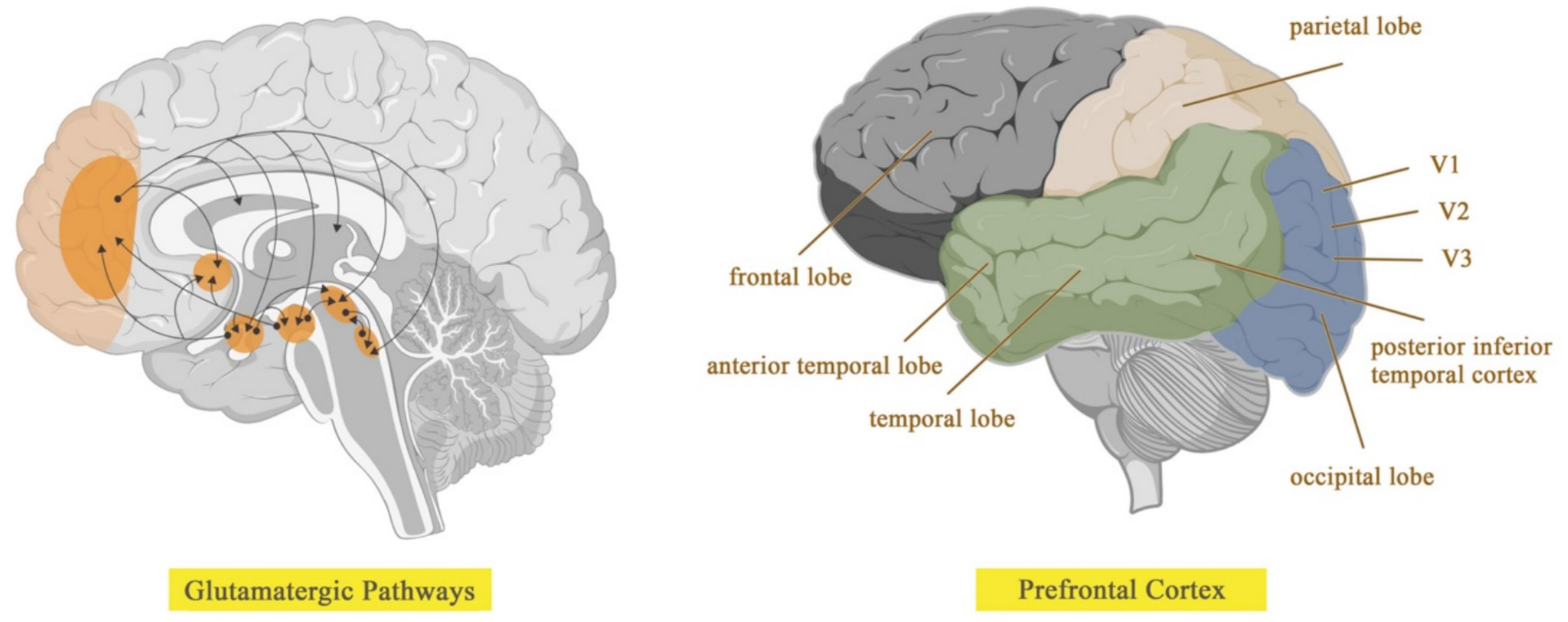
The cerebral cortex is functionally partitioned.

Gender differences are significant in the mental perception of color, which is closely related at the physiological level to patterns of neural activity in the visual cortex. Among the 16 visual regions of the brain that influence color perception in humans, it is the human visual cortex (Ogle, 1951; Fabius et al., 2022) including the initial visual cortex (Troncoso et al., 2015), the striate cortex (Stoerig, 2006; Daddaoua et al., 2014) and the extrastriate cortex (Graziano et al., 1994; Li et al., 2022) (Figure 8). Physiological differences between males and females significantly influence their color preferences. In men, neural activity in the parietal region of the brain is lateralized to the right hemisphere when evaluating personal preferences, placing more emphasis on holistic features. In contrast, women have activity in both parietal lobes, integrating both holistic and local features in aesthetic assessments (Cela-Conde et al., 2009).

Recently, there has been a steady increase in the number of studies on color preferences influenced by gender, with 23 relevant articles published in the past decade. More attention is being paid to the perceptions and preferences of different groups of people in urban environments. Guilford and Smith were among the earliest scholars to examine the influence of gender on spatial perception, proposing that women tend to be more sensitive to spatial color change (Guilford and Smith, 1959). Middle-aged adult women tend to prefer warm colors over men, primarily due to the emotional and psychological comfort needs associated with warm hues (Hoxha et al., 2023). This difference is widely found in the choice of urban colors, such as room colors (Iheme et al., 2018). There is also a strong link between personality traits and psychological perceptions of color. Extroverts generally favor warm colors with high brightness and saturation that stimulate positive emotions and higher levels of arousal, while introverts prefer cool colors with low brightness and saturation for psychological peace and security (Choungourian, 1967). Personality traits were significantly correlated with color preference for different occupational groups (Rosenbloom, 2006). Individuals with creative jobs preferred red, while those in more technical roles tended to favor blue.

As the aging process accelerates within society, research on individual color preferences has increasingly shifted toward the creation of color environments adapted to the elderly. The development of age-appropriate built environments has thus become a central focus of research and a key objective of sustainable development (Jaglarz, 2024).

**Figure 9:**
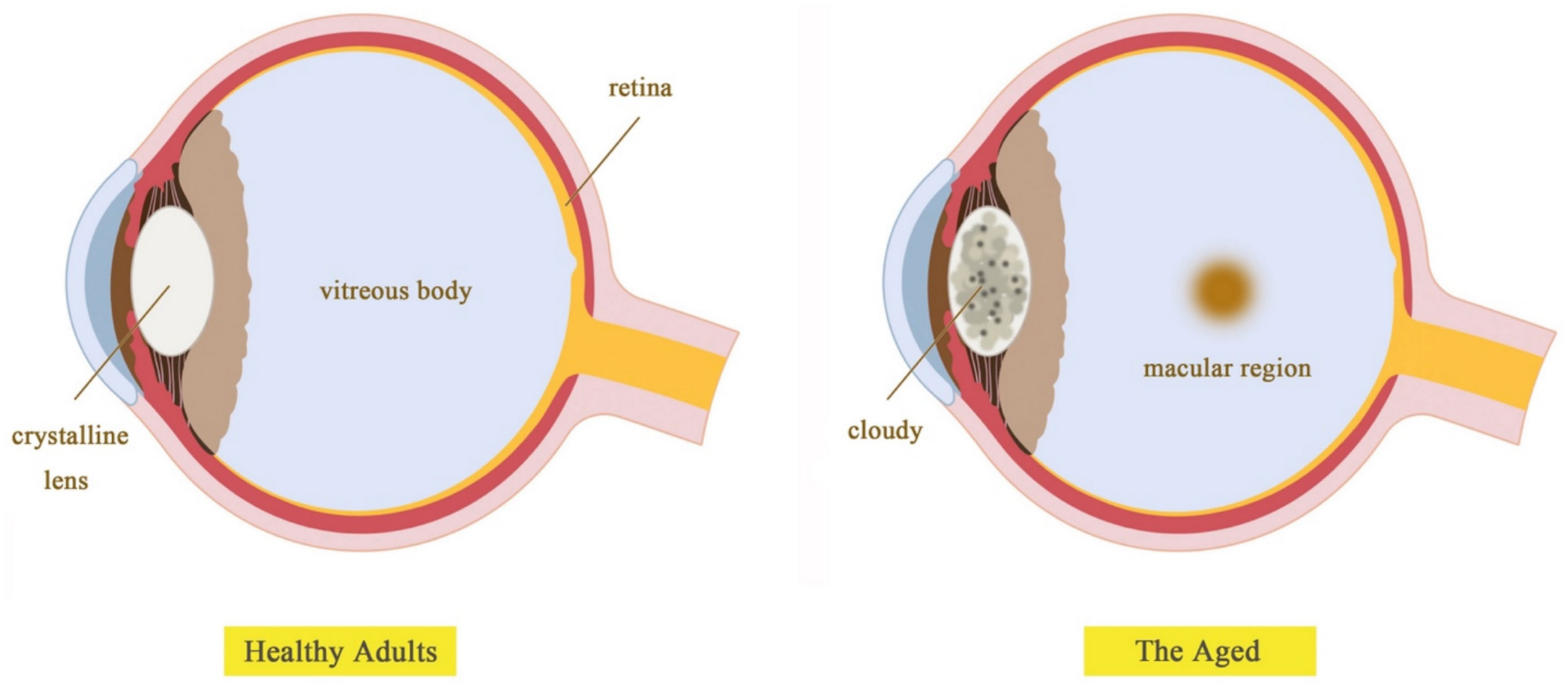
Depicts the typical lens of an adult and an elderly individual.

Aging can alter an individual’s color perception of the environment due to changes in the spectral properties of light received by the retina in older adults (Xu et al., 1997; Silva et al., 2021). These changes include the gradual loss of retinal ganglion cells and their axons (Pearson et al., 2006; Harwerth et al., 2008), as well as alterations in the lens’s absorption of short-wavelength light (Hensch, 2005; Zelentsova et al., 2017). Additionally, psychological factors, such as declines in cognitive processing (Ou et al., 2012), contribute to a different perception of color compared to younger individuals (Figure 9).

There is a significant link between personality traits, mental states, and psychological perceptions of color. Additionally, a strong relationship exists between emotions and color sensitivity (Barrick et al., 2002). Extroverts usually favor warm colors with high brightness and saturation that stimulate positive emotions and higher levels of arousal, while introverts prefer cool colors with low brightness and saturation for psychological peace and security (Choungourian, 1967). Depression-prone individuals prefer low-color, low-lightness colors (Eysenck and Keane, 2020), while mentally healthy, emotionally positive people tend to prefer warm tones (Wu et al., 2009). Additionally, color influences the perception of pain (Wiercioch-Kuzianik and Bąbel, 2019). Depressed, abused, and chronically schizophrenic individuals often associate red and black hues with pain and fear (Jue and Kwon, 2013).

This offers enhanced color design strategies for medical and convalescent spaces, linking color to a specific location, and using people’s different preferred colors as a placebo to soothe groups of anxious or frightened people to achieve the effect of visual therapy (McLachlan and Leng, 2021). For instance, patients with mental illnesses tend to prefer environments featuring natural colors, such as those found in ocean waves, rocks, and snowfields (Lengen, 2015). Labor and delivery spaces are typically designed with a softer color palette to alleviate the stress of childbirth and enhance the overall quality of care (Duncan, 2011).

### 4.2 Sociocultural influences and emotional preferences

**Figure 10:**
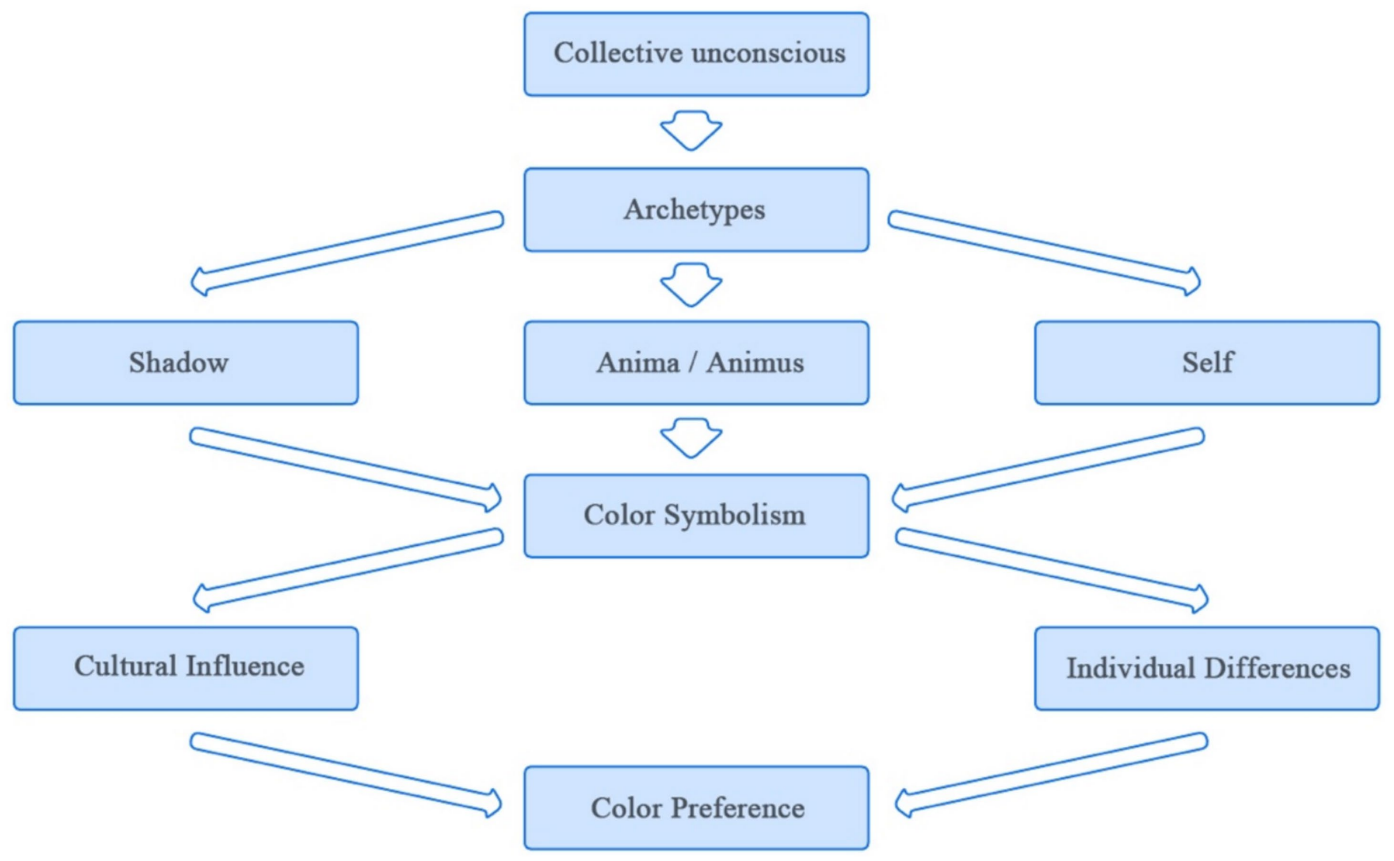
Effect of collective unconscious on color preference.

The socio-cultural influence on color preference has been a marked subject of research in the field. Ethnic customs, religious beliefs, and regional lifestyles are often reflected in urban spaces using color symbolism (Tarajko-Kowalska, 2023). Carl Jung’s theory of the collective unconscious (McKimm, 2017) highlights the relationship between color perception and emotional responses across various domains of experience (Figure 10). Many of an individual’s preferences and behaviors are influenced by unconscious factors, including childhood experiences, underlying emotions, and deep psychological needs.

Colors associated with specific social symbols and meanings (Oberascher and Oberascher, 2002; Jonauskaite et al., 2019a) in human societies are influenced by both semi-conscious factors (Al-Rasheed, 2015), such as learning and cultural influences, and conscious factors (Motamed and Tucker, 2019), including personal experience, personality, popular trends, contemporary culture, and social semiotics. Consequently, the interpretations of the emotional associations and symbolic meanings of specific colors in different contexts often vary significantly.

Humphrey’s evolutionary theory suggests that color preference is influenced by signals transmitted by natural organisms (Humphrey, 2019), with scarcity serving as a significant factor that shapes and directs color preference. Psychological ownership is further strengthened when individuals are exposed to scarce colors. In environmental spaces, scarce colors are considered more valuable resources in primitive societies and are attributed higher emotional and symbolic significance compared to the more common, universal colors present in everyday life (Williams et al., 2024). Religious sites and government institutions frequently utilize colors to signify power and social hierarchy (Wu et al., 2018).

In traditional societies, the use of color symbolism is primarily driven by three key factors: economic, historical, and social (Hutchings, 2004). Urban color preferences are not only closely linked to the aesthetic concepts of local cultures but are also profoundly shaped by social and historical symbol systems. Cultural units or cultural memes (Kelly and De Block, 2022), shared across different ethnic groups, significantly influence their color perception systems.

Different national patterns also influence color preferences, which are often reflected in cross-cultural color symbolism and emotional associations. Individuals from modern collectivist societies tend to show more significant convergence in color preferences than people from modern individualistic societies due to their high level of interdependence (Oyserman and Lee, 2008; Arpaci et al., 2018). Individualism and collectivism, as two extreme forms of self-construction, extend to behavioral norms, values, patterns of social interaction, and ways of self-definition in addition to influencing an individual’s preference for environmental traits (Brewer, 1991), but people from the same culture will share similar values (Zeng et al., 2024), and associative couplings of colors are ingrained in cultural practices (Najar et al., 2024) and become part of cultural identity. This also includes the similarity of color associations. For instance, individuals in Islamic cultures tend to prefer dark and neutral shades, which are deeply rooted in their religious and cultural traditions (Motamed and Tucker, 2019). In many Asian cultures, red is regarded as a symbol of good fortune and celebration, which is why it is prominently used during festive occasions. In western cultures, blue is often linked to a sense of trust and stability, making it a popular choice in the design of public and religious buildings (Joshi and Rawat, 2020).

With the advancement of globalization, urban color preferences have evolved in the context of cross-cultural exchanges. In multicultural communities, variations in color perception and preferences among residents from diverse ethnic and cultural backgrounds (Cao et al., 2023) must be carefully considered. Recognizing these differences can foster a stronger sense of belonging and security among residents (Wang and Cao, 2022), ultimately contributing to the development of distinctive place-attachment emotions.

### 4.3 Environmental space and color psychological tendencies

The influence of landscape context on urban color preferences is particularly significant. The environment not only directly influences color perception at the visual level but also deeply affects individuals’ psychological needs, emotional responses, and behavioral patterns. Environmental Behavior Theory (EBT) explores the interaction between the environment and human behavior, especially behavioral patterns in social spaces (Yuriev et al., 2020). According to EBT theory, environmental design not only influences individuals’ emotions and perceptions, but also subliminally guides and shapes behaviors (Sawitri et al., 2015).

**Figure 11:**
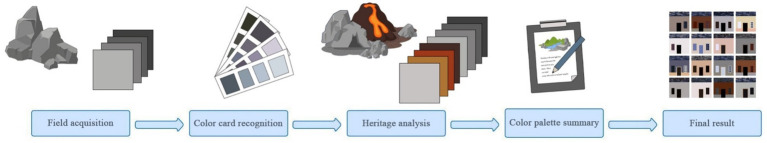
The concept and application processes of color geography.

From the perspective of color geography (Lenclos and Lenclos, 2004) research, the natural geographic environment is not only the foundation of human society (Wang, 2017) but also an important factor affecting the choice of urban color (Figure 11). People living in countries far from the equator, often characterized by cold and rainy climates (e.g., Finland), tend to prefer yellow due to a desire for warmth and sunlight (Jonauskaite et al., 2019a). In contrast, in desert regions like Egypt, yellow is considered an ominous symbol (Azer, 2020). In the traditional architecture of ethnic minorities in the Indian Himalayas, the use of blue-white colors has come to symbolize cultural identity, influenced by socio-geographical factors (Joshi and Rawat, 2020).

Environmental characteristics such as landscape vegetation, climatic conditions, and architectural styles in urban areas directly influence residents’ color perception and preferences (Li and Cao, 2022). The colors present in the landscape are closely linked to the human circadian rhythm. Research indicates that exposure to natural environments (e.g., green spaces and blue spaces) enhances mental health (Michels et al., 2022), reduces stress, promotes well-being, and increases life satisfaction (Kotera et al., 2021). These natural colors, often referred to as “life colors” (Zheng et al., 2024), can unconsciously evoke positive emotional responses in individuals.

From a psychological perspective, blue and green are the most influential colors. Blue, derived from natural elements such as the sky, the sea, and snow-capped mountains, symbolizes depth and mystery. Despite varying geographical contexts, the color blue holds significant symbolic associations across many cultures, often linked to concepts such as the divine, truth, and peace (Tarajko-Kowalska, 2023). As a cool color, blue arouses feelings of calmness, coolness, and relaxation, which makes it especially effective in environments such as public spaces, offices, and healthcare environments. In the 1960s, as architects incorporated green into urban planning and design, green landscapes significantly influenced the color preferences of city dwellers. Green is associated with both psychological and ecological benefits, reflecting a deep-seated human pursuit of nature and a healthy lifestyle. Positive interactions in green spaces increase individuals’ attraction to them (Paraskevopoulou et al., 2018). Especially for individuals experiencing psychological trauma, the introduction of green helps in emotional healing and recovery.

In addition to natural landscapes, architectural colors in urban spaces play a significant role in shaping individuals’ psychological and emotional responses. With the trend of times and technological progress, changes in urban architectural styles are often accompanied by continuous shifts in color selection. The choice of architectural colors across different eras not only reflects societal aesthetic demands but also reflects the collective psychology of a specific historical period and social background. For instance, the popularity of modern minimalist styles prompted the widespread use of calm tones such as gray and white in urban architecture, symbolizing the arrival of modernism (Serra and Codoñer, 2014). In the development and renewal of urban spaces, colors influence people’s preferences through symbolism shaped by historical events, cultural traditions, and religious beliefs (Motamed and Tucker, 2019).

With the advancement of urban renewal and sustainability, environmentally friendly color palettes are increasingly applied in urban revitalization projects. Modern tactical urbanism advocates a low-cost approach to color transformation, leveraging an open, iterative development process and the creative potential fostered by social interaction to enhance urban attractiveness and strengthen residents’ sense of belonging through strategic color application (O’Connor, 2023). For instance, in the design of urban streets, public squares, and residential areas, color is employed to demarcate sidewalks, green spaces, and social zones, thereby improving safety initiatives, enhancing pedestrian accessibility, and enriching urban aesthetics (O’Connor, 2021). Urban planners must consider the psychological impact of environmental design to regulate emotions, enhance spatial comfort, and reinforce residents’ sense of belonging.

### 4.4 Dynamic factors and bidirectional feedback efficiency

Although dynamic factors have not been extensively studied in the existing literature, research interest in this area has continued to increase in recent years. The dynamic nature of urban color preferences is influenced by seasonal variations, the process of urbanization, social transformations, and technological advancements, all of which exhibit discernible patterns of change.

Color preferences dynamic color preferences not only reflect changes in the social environment but are also closely related to ecological benefits. Ecological Value Theory (EVT) suggests that mental imagery also influences individuals’ color preferences (Palmer and Schloss, 2010). Individual color preferences are influenced by the emotional experience of color-related entities and adjust dynamically with their positive or negative experiences (Schloss et al., 2011).

Individuals typically prefer colors associated with favored objects (e.g., the blue of the sky and lakes) and avoid those linked with disliked objects (e.g., the brown of rotting food). Such unconscious preferences based on memory associations can vary depending on individual experience (Schloss and Palmer, 2017), for example, green appears negative when associated with pond mold but positive when associated with fresh vegetables, which in turn affects attention, affective responses, and cognitive processes.

Since the introduction of EVT, the number of studies exploring color preferences within this theoretical framework has steadily increased. To assess the accuracy of EVT, a Weighted Affective Valence Estimation (WAVE) model was further developed (Palmer and Schloss, 2010). The model calculates the average emotional value of individuals’ responses to objects associated with each color, weighted by the strength of association with that color. Where each color is represented by *c*, the average valence is *v_o_*, the object associated with the color is *o*, and the object-color match is *w_co_*, the formula is as follows:


$$\mathrm{Wc}=\frac{1}{n_{c}}\sum_{o=1}^{n_{c}}w_{\mathrm{co}}v_{o}$$

The significant influence of ecological factors on human color preferences can be illustrated through the WAVE model. The strength of the object-color association accounts for nearly 80% of the variance in the factors influencing color preferences among western adults (Palmer and Schloss, 2010; Al-Rasheed et al., 2022), thereby explaining the average pattern of color preferences.

Seasonal changes are also a significant dynamic factor influencing color preferences. Individuals tend to favor deep warm tones during autumn (Schloss et al., 2017), while climatic variations lead to a preference for warm colors (e.g., orange, red) that bring a sense of visual warmth in colder regions (Azer, 2020). In contrast, cooler tones (e.g., blue, green) are more common in tropical regions. Therefore, dynamic urban color design that incorporates seasonal characteristics can help regulate the urban atmosphere, enhancing both spatial experience and comfort.

Changes in dynamic factors are also reflected in rapid social, cultural, and technological changes. Modern urbanization is frequently accompanied rapid changes in architectural styles and functions, which drives the diversification and renewal of urban color choices (Odetti, 2023). With society’s growing focus on health and ecology, urban color choices are increasingly gravitating toward hues that enhance the well-being and quality of life for residents (Xu and Zheng, 2021).

The popularization and application of green buildings and environmentally friendly materials have led to a tendency to use green and natural tones in urban architectural design to meet the needs of current development (Xu and Zheng, 2021). These changes not only help to improve the functionality and sustainability of urban spaces (Guo et al., 2025), but also effectively regulate the emotional experience of residents and promote the overall improvement of urban quality of life.

## 5 Conclusion

Based on a bibliometric analysis of 379 studies, this research systematically examines the current state, research hotspots, and future trends related to the factors influencing color preference in urban environments. The results show that the formation of urban color preferences is a complex psychological process shaped by individual differences, socio-cultural factors, environmental contexts, and broader dynamic forces.

This study contributes to urban design and color theory by offering a comprehensive understanding of the mechanisms behind color preference. It highlights how personal attributes—such as age, gender, emotional memory, and lifestyle—play a key role in shaping individual responses to color. Emotional memory, in particular, enriches the diversity of preferences by associating specific hues with lived experiences. Sociocultural influences also moderate color preferences, with significant variations observed across different regions, religious traditions, and historical contexts. In addition, the design of environmental space—reflected in natural landscapes, architectural styles, and urban functions—is closely aligned with psychological needs, often favoring colors such as green and blue for their calming effects. Furthermore, trends such as globalization, urbanization, and technological advancement are transforming color preferences through cross-cultural integration and the growing adoption of natural hues in sustainable architecture. These findings underscore the role of dynamic color management in enhancing both aesthetic quality and residents’ psychological well-being.

The findings offer practical insights for urban planners and designers seeking to create inclusive, culturally sensitive, and emotionally supportive spaces. By addressing diverse user needs through informed color strategies, cities can improve not only their visual identity but also the everyday experiences of their inhabitants.

Despite its contributions, this study has certain limitations. The analysis relies solely on existing literature, potentially omitting emerging research or non-English sources. Moreover, the conclusions are drawn from secondary data and lack empirical validation. Future research should adopt empirical, mixed-method, and cross-cultural approaches to strengthen the generalizability and practical relevance of the findings.

Building upon these insights, future studies are encouraged to explore new directions. First, the dynamic effects of seasonal changes and light cycles on color perception remain underexplored. Combining immersive technologies such as virtual reality with real-time environmental data could offer a more nuanced understanding of temporal color preferences. Second, the integration of big data analytics and machine learning models holds potential for accurately predicting individual and group-level color preferences, enabling more personalized and adaptive urban design. Interdisciplinary research can further uncover the cognitive and emotional mechanisms through which color influences behavior, providing a holistic understanding of its role in shaping urban life.

## Data Availability

The original contributions presented in the study are included in the article/supplementary material, further inquiries can be directed to the corresponding author.
